# Immune checkpoint inhibitors in breast cancer: development, mechanisms of resistance and potential management strategies

**DOI:** 10.20517/cdr.2023.58

**Published:** 2023-11-17

**Authors:** Rachel SJ Wong, Rebecca JM Ong, Joline SJ Lim

**Affiliations:** ^1^Department of Haematology-Oncology, National University Cancer Institute, National University Hospital, Singapore 119228, Singapore.; ^2^Yong Loo Lin School of Medicine, National University of Singapore, Singapore 119228, Singapore.

**Keywords:** Immunotherapy, immune checkpoint inhibitors, resistance mechanisms, breast cancer

## Abstract

The use of immune checkpoint inhibitors (ICIs) has increased exponentially in the past decade, although its progress specifically for breast cancer has been modest. The first U.S. Food and Drug Administration approval for ICI in breast cancer came in 2019, eight years after the first-ever approval of an ICI. At present, current indications for ICIs are relevant only to a subset of patients with triple-negative breast cancer, or those displaying high microsatellite instability or deficiency in the mismatch repair protein pathway. With an increasing understanding of the limitations of using ICIs, which stem from breast cancer being innately poorly immunogenic, as well as the presence of various intrinsic and acquired resistance pathways, ongoing trials are evaluating different combination therapies to overcome these barriers. In this review, we aim to describe the development timeline of ICIs and resistance mechanisms limiting their utility, and summarise the available approaches and ongoing trials relevant to overcoming each resistance mechanism.

## INTRODUCTION

Breast cancer is the most common cancer worldwide, accounting for 12.5% of all new cancer cases globally, and is the leading cause of cancer mortality in women^[1]^. In the year 2020, an estimated 2.3 million female breast cancers were diagnosed globally, and about 685,000 women died from their disease^[2]^. This number is expected to grow to more than 3 million new cases diagnosed and 1 million deaths by the year 2040^[2]^.

With advances in our understanding of cancer biology, immuno-oncology has become an area of great interest and extensive research. Cancer immunotherapy employs the use of cutting-edge technologies, including immune checkpoint inhibitors (ICIs) such as those targeting Programmed Cell Death Protein-1 (PD-1), Programmed Cell Death Ligand-1 (PD-L1), and Cytotoxic T-Lymphocyte-Associated Antigen 4 (CTLA-4), and more recently, chimeric antigen receptor (CAR) T cell therapies. Other frontiers being pushed in the realms of immunotherapy include the use of cancer vaccines^[3]^, for cancer prevention, such as vaccines for Human Papilloma Virus and Hepatitis B^[4]^, as well as in cancer treatment, as in the case of Sipuleucel-T for prostate cancer^[5]^.

Since the first U.S. Food and Drug Administration (FDA) approval of ipilimumab, a CTLA-4 monoclonal antibody, in 2011 for the treatment of metastatic melanoma^[6]^, ICIs have transformed the treatment landscape across multiple tumour types^[7]^. There are now eleven FDA approvals for ICIs: two CTLA-4 inhibitors (ipilimumab, tremelimumab), five PD-1 inhibitors (pembrolizumab, nivolumab, cemiplimab, dostarlimab, retifanlimab), three PD-L1 inhibitors (atezolizumab, avelumab, durvalumab), and one lymphocyte-activation gene 3 (LAG-3) blocking antibody (retatlimab)^[6-9]^. However, amongst the numerous available approvals for ICIs, there are currently only two specific FDA approvals in the setting of breast cancer, both for pembrolizumab in the subgroup of triple-negative breast cancer (TNBC), given in combination with chemotherapy in the metastatic^[10]^ and neoadjuvant^[11]^ settings. Additional FDA approvals that are tumour agnostic and apply to breast cancer include pembrolizumab^[12]^ and dostarlimab^[13]^ in breast cancers displaying high microsatellite instability (MSI-H) or a deficiency in the mismatch repair protein (dMMR) pathway. While one of the hallmarks of treatment with ICIs is its durable response that translates to prolonged survival of these patients, admittedly, only a very small subset of patients benefit. In this review article, we will first describe the evolution of ICI in the TNBC subtype, focusing on its approved indications, before delving into the understanding of the resistance mechanisms towards ICIs, and how we can harness such knowledge to develop new combination strategies.

## EVOLUTION OF ICIS IN BREAST CANCER

### Monotherapy ICIs in TNBC

Evidence for the use of ICIs in breast cancer first came from single-agent immunotherapy trials in the metastatic setting, including the KEYNOTE-012 and KEYNOTE-086 studies. KEYNOTE-012 was a phase I study that aimed to evaluate the role of single-agent pembrolizumab in patients with various advanced solid tumours. In the cohort of TNBC who had progressed on a median of 2 lines of treatment, the objective response rate (ORR) was 18.5% and 6-month progression-free survival (PFS) was 24.4%, 6-month and 12-month overall survival (OS) were 66.7% and 43.1%, respectively^[14]^. The investigators observed that there was a suggestion of response with increasing expression of PD-L1, albeit within a small sample size (*n* = 32).

KEYNOTE-086 was designed specifically to look at the role of pembrolizumab monotherapy in patients with metastatic TNBC. This phase II multicohort study included all comers with ≥ 1 prior systemic treatment for metastatic disease regardless of PD-L1 status (Cohort A)^[15]^, and also patients with no prior systemic treatment in the metastatic setting who had PD-L1 positive tumours defined as combined positive score (CPS) ≥ 1 based on the Dako PD-L1 IHC 22C3 platform (Cohort B)^[16]^. Comparing across cohorts, there was a suggestion of improved ORR in less heavily pre-treated patients (ORR 21.4% *vs.* 5.3%) in Cohort B *vs.* all-comers in Cohort A. This was consistent with other similar phase 1 trials evaluating avelumab (JAVELIN study)^[17]^ and atezolizumab (PCD4989g trial)^[18]^ as monotherapy in metastatic TNBC, suggesting clinical benefit when used in earlier lines of treatment and PD-L1 expressing tumours.

A subsequent KEYNOTE-119 randomised phase III trial compared pembrolizumab monotherapy *vs.* single agent physicians’ choice chemotherapy in patients who progressed on 1 or 2 prior lines of treatment for metastatic TNBC^[19]^. While the trial was negative for its primary endpoint of OS in all subgroups, there was a positive trend to survival benefit in patients with PD-L1 CPS ≥ 10 (12.7 *vs.* 11.6 months, HR 0.78; *P* = 0.057).

The limited efficacy of single-agent immunotherapy observed in breast cancer might be due to intrinsic tumour resistance due to its complex and enigmatic relationship with the immune system. Breast cancer was traditionally thought to be poorly immunogenic, also known as a “cold tumour”. Immunogenicity, or the ability to elicit an antitumoural response by the body’s immune system, is dependent upon the formation of neo-antigens that are derived from gene mutations, viral oncogenes alternative splicing, or gene rearrangement^[20-22]^. It is assessed by the antigenicity of a cancer, which in turn is evaluated by its mutagenicity^[23]^. One measure of the antigenicity of cancer is its mutational load or tumour mutational burden (TMB), which refers to the average number of somatic mutations per (Mb)^[23,24]^. Cancers like melanoma and lung cancer are known to be “hot tumours”, as observed in a study by Chalmers *et al.* who reported their median TMB levels to be 13.5 mut/Mb and 7.2 mut/Mb, respectively^[25]^. In contrast, the TMB in breast cancer is generally much lower. In a study by Barroso-Sousa *et al.*^[26]^ of 3,969 patients with breast cancer, the median TMB reported was 2.63 mut/Mb, while another Chinese study of 196 breast cancer patients demonstrated a higher median TMB of 4.03 mut/Mb^[27]^. Due to the poor efficacy observed with the use of single-agent immunotherapy treatment, further efforts were directed at exploring combination treatment.

### Combining ICIs with chemotherapy in TNBC

The rationale for combination treatment with chemotherapy was that chemotherapeutic agents had been shown to have synergistic effects with ICIs by inducing immunogenic cell death, causing the release of tumour-associated neoantigens as well as its ability to stimulate immune surveillance^[28,29]^. Indeed, this has proven to be an effective strategy in several subgroups of TNBC.

The initial results of several phase I studies evaluating this combination in the setting of metastatic TNBC were promising, reporting response rates ranging between 23.4%-39%^[30,31]^. Several phase III trials confirmed these positive preliminary findings, leading to the first FDA-approved indication for an ICI for use in breast cancer treatment.

#### Atezolizumab

The first FDA accelerated approval of an ICI for breast cancer was with the anti-PD-L1 inhibitor atezolizumab, which was granted on 8 March 2019^[32]^ based on the IMpassion 130 trial^[33]^. This phase III placebo-controlled randomised trial evaluated 902 patients with treatment naïve, unresectable locally advanced or metastatic TNBC. Patients were randomised to receive either atezolizumab or its placebo, in combination with albumin-bound paclitaxel (nab-paclitaxel). In patients whose tumours expressed PD-L1 based on the VENTANA PD-L1 SP142 assay, there was a significant median PFS benefit: 7.5 months in patients receiving atezolizumab *vs.* 5.0 months with placebo (HR 0.62, *P* < 0.001). The final approval of this combination was contingent upon the results of the IMpassion 131 trial evaluating atezolizumab with paclitaxel in TNBC in the same setting, which unfortunately failed to meet its primary endpoint of superior PFS^[34]^. This led to Genentech voluntarily withdrawing the previously granted accelerated FDA approval for atezolizumab on 27 August 2021. Eventually, when the final OS was read out for the IMpassion 130 trial, the addition of atezolizumab to nab-paclitaxel failed to meet statistical significance, precluding further testing^[35]^.

#### Pembrolizumab

Pembrolizumab is currently FDA-approved for use in TNBC in the first-line metastatic and neoadjuvant settings, both in combination with chemotherapy. It first received FDA approval on 13 November 2020 as combination therapy with chemotherapy for patients with unresectable locally-advanced or metastatic TNBC whose tumours have a PD-L1 CPS ≥ 10 based on the Dako 22C3 assay^[10]^. This was based on KEYNOTE-355, a phase III randomised placebo-controlled study evaluating the role of pembrolizumab in combination with chemotherapy in patients in the above-mentioned setting. It reported a median OS (mOS) benefit of about 7 months in patients whose tumours expressed PD-L1 CPS ≥ 10 (mOS 23.0 *vs.* 16.1 months; HR 0.73, *P* = 0.0185). In patients whose tumours expressed PD-L1 CPS ≥ 1 or in the intention-to-treat population, there was no survival benefit shown.

In addition, pembrolizumab also has tumour-agnostic FDA approval for advanced unresectable or metastatic solid tumours that are dMMR or MSI-H^[12]^. This was based on the combined results of 5 single-arm trials where a total of 149 patients with dMMR/MSH-H solid tumours achieved an ORR of 39.6%, with 78% of patients having responses lasting 6 months or more. It should be noted, however, that only 2 out of the 149 patients had breast cancer. They both achieved partial responses, with duration of response (DoR) of 7.6 and 15.9 months^[36]^.

#### Dostarlimab

Most recently, on 17 August 2021, dostarlimab also received accelerated FDA approval for recurrent or advanced solid tumours that are dMMR based on the GARNET trial^[13]^. This was an open-label, non-randomised, multicohort phase I trial evaluating dostarlimab as monotherapy in the above-mentioned clinical setting. In these patients, there was an ORR of 41.6%, with 9.1% complete responses and 32.5% partial responses. The median DoR was 34.7 months, with 95.4% of patients still showing continued response at 6 months. In cohort F, which enrolled 106 non-endometrial solid tumours, 1 patient had dMMR breast cancer and reported a complete response^[37]^.

With the promising results of a combination of ICI therapy and chemotherapy in the metastatic setting, efforts were then shifted to study it in the earlier curative stages of breast cancer. One of these trials is the phase II I-SPY 2 trial, which adopted an adaptive trial design to evaluate various novel therapeutics in combination with chemotherapy, comparing that to standard treatment as in the neoadjuvant setting for early-stage breast cancer^[38]^. Pembrolizumab was included in one of the study arms, where patients were randomised to receive 4 cycles of pembrolizumab given in combination with weekly paclitaxel *vs.* weekly paclitaxel alone, followed by doxorubicin/cyclophosphamide and then definitive surgery. Compared to standard chemotherapy alone, the addition of pembrolizumab improved pathologic complete response (pCR) rates in all breast cancer subtypes: 44% *vs.* 17% in HER2-negative breast cancers, 30% *vs.* 13% in HR-positive/HER2-negative breast cancers, and 60% *vs.* 22% in TNBC^[39]^.

Focusing on the TNBC subtype, the role of pembrolizumab in the neoadjuvant setting was proven in the confirmatory phase III KEYNOTE-522 trial, which subsequently led to pembrolizumab receiving its second breast cancer-specific FDA approval on 26 July 2021^[11]^. In this phase III randomised controlled study, 1,174 patients with previously untreated stage II or III TNBC were randomised in a 2:1 ratio to receive pembrolizumab or a placebo, respectively, in combination with chemotherapy , before undergoing surgery. Pembrolizumab or its placebo was continued post-operatively for up to 9 cycles. Both primary endpoints of the trial were met; there was a significant improvement in pCR of 64.8% *vs.* 51.2%; *P* = 0.00055, although this had reduced by the third interim analysis^[40]^ to 63.0% *vs.* 55.6%. There was also an improvement in 3-year event-free survival (EFS) 84.5% *vs.* 76.8%; *P* < 0.001^[41]^. Interestingly, contrary to data in the metastatic setting, PD-L1 expression was not predictive of benefit^[11]^, and consequently the FDA approval in the neoadjuvant setting was granted irrespective of PD-L1 expression.

The benefit of ICIs in combination with chemotherapy in the neoadjuvant setting was also echoed in the IMpassion 031 study evaluating atezolizumab. In IMpassion 031, atezolizumab was evaluated in the neoadjuvant setting in patients with stage II-III TNBC treated for curative intent. This was a double-blind phase III randomised trial where patients received either atezolizumab or its placebo, in combination with nab-paclitaxel, followed by doxorubicin and cyclophosphamide. The investigators found an increase in pCR rates from 58% *vs.* 41% in the all-comers population; *P* = 0.0044, (significance boundary 0.0184), and 69% *vs.* 49% in PD-L1 positive patients; *P* = 0.021, (significance boundary 0.0184). As it did not hit the prespecified boundary of significance for its second co-primary endpoint, the study is not formally powered for further survival analyses^[42]^.

However, not all trials evaluating the addition of ICIs in combination with chemotherapy in the neoadjuvant setting for TNBC have yielded similar results. Both the NeoTRIP and GeparNeuvo evaluating atezolizumab and durvalumab, respectively, in the neoadjuvant setting were negative for pCR benefit. Patients in the NeoTRIP study were randomised to receive neoadjuvant carboplatin and nab-paclitaxel with or without 8 cycles of atezolizumab. Anthracyclines were given in the adjuvant setting after definitive surgery. The addition of atezolizumab resulted in numerically higher but nonsignificant pCR rates: 48.6% *vs.* 44.4%; *P* = 0.48^[43]^. Similarly, the GeparNuevo trial studied the addition of durvalumab to neoadjuvant chemotherapy with paclitaxel followed by epirubicin and cyclophosphamide, which found a nonsignificant but numerically superior pCR rates of 53.4% *vs.* 44.2%; *P* = 0.224^[44]^. Interestingly, a survival benefit with the addition of durvalumab compared to placebo was observed; 3-year invasive disease-free survival (iDFS) was 84.9% *vs.* 76.9% (HR 0.54; *P* = 0.0559) and 3-year OS 95.1% *vs.* 83.1% (HR 0.26; *P* = 0.0076)^[45]^.

While there is general consensus for the use of ICIs in combination with chemotherapy in the neoadjuvant setting for TNBC, its optimal duration is currently still widely discussed. In both KEYNOTE-522 and IMpassion 031, the ICI was continued post-operatively for a total of 1 year, while NeoTRIP and GeparNuevo only administered ICI in the neoadjuvant setting. GeparNuevo is the only study that has shown survival benefits with the use of ICI despite being administered only in the neoadjuvant context without continuation in the adjuvant setting, leading to questions of whether there is a need for continual ICI in the adjuvant setting. Additionally, the pCR benefit that was observed in the durvalumab group in GeparNuevo was exclusively seen in the cohort of patients who received a 2-week lead-in of durvalumab prior to chemotherapy, although the reason for this observation is currently unclear. We have summarised the trials evaluating the use of ICI both as monotherapy and in combination with chemotherapy in Table 1.

**Table 1:** Summary of trials evaluating the use of ICI as monotherapy and in combination with chemotherapy

**Trial name/ID**	**Phase**	**Population**	**Arms**	**Results**
KEYNOTE-012 NCT01848834	I	Advanced TNBC, PD-L1 + ve; pre-treated	Pembrolizumab	ORR 18.5% 6-mo PFS 24.4% 6-mo OS 66.7%, 12-mo OS 43.1%
KEYNOTE-086 NCT02447003	II	Metastatic TNBC; pre-treated Cohort A: all-comers Cohort B: PD-L1 + ve	Pembrolizumab	Cohort A: ORR 5.3%, mPFS 2.0 mo, mOS 9.0 mo Cohort B: ORR 21.4%, mPFS 2.1 mo, mOS 18.0 mo
JAVELIN NCT01772004	I	Metastatic breast cancer; pre-treated	Avelumab	ORR: 3.0% (overall population), 5.2% (TNBC), 16.7% (PD-L1 + ve), 1.6% (PD-L1-ve)
PCD4989g NCT01375842	I	Metastatic TNBC; any-line	Atezolizumab	ORR 24% (1st line), 6% (≥ 2nd line) ORR 12% (1st line), 0% (≥ 2nd line) mOS 10.1 mo (PD-L1 + ve), 6.0 mo (PD-L1-ve)
KEYNOTE-119 NCT02555657	III	Metastatic TNBC; 1 or 2 prior lines	Pembrolizumab *vs.* chemotherapy	mOS 9.9 mo *vs.* 10.8 mo HR 0.97 (overall population) mOS 12.7 mo *vs.* 11.6 mo HR 0.78; *P* = 0.057 (PD-L1 CPS ≥ 10)
IMpassion 130 NCT02425891	III	Metastatic TNBC; untreated	Nab-paclitaxel +/- atezolizumab	mPFS 7.2 mo *vs.* 5.5 mo, HR 0.79; *P* = 0.002 (ITT) mOS 21.0 mo *vs.* 18.7 mo HR 0.87; *P* = 0.077 (ITT) mPFS 7.5 mo *vs.* 5.0 mo HR 0.63, *P* < 0.0001 (PD-L1 + ve) mOS 25.4 mo *vs.* 17.9 mo HR 0.67; (PD-L1 + ve)
IMpassion 131 NCT03125902	III	Metastatic TNBC; untreated	Paclitaxel +/- atezolizumab	mPFS 6.0 mo *vs.* 5.7 mo, HR 0.82; *P* = 0.20 (PD-L1 + ve) mPFS 5.7 mo *vs.* 5.6 mo, HR 0.86 (ITT)
KEYNOTE-355 NCT02819518	III	Metastatic TNBC; untreated	Chemotherapy +/- pembrolizumab	mPFS 9.7 mo *vs.* 5.6 mo HR 0.66 (CPS ≥ 10) mPFS 7.6 mo *vs.* 5.6 mo HR 0.75 mOS 23.0 *vs.* 16.1 mo HR 0.73; *P* = 0.0185 (CPS ≥ 10) mOS 17.6 mo *vs.* 16.0 mo HR 0.86 *P* = 0.1125 (CPS ≥ 1)
I-SPY 2 NCT01042379	II	High-risk stage II/III breast cancer	Chemotherapy +/- pembrolizumab	pCR 44% *vs.* 17% (ERBB2-negative), 30% *vs.* 13% (HR- + ve/ERBB2-ve), 60% *vs.* 22% (TNBC)
KEYNOTE-522 NCT03036488	III	Stage II/III TNBC	Chemotherapy +/- pembrolizumab	pCR 64.8% *vs.* 51.2%; *P* = 0.00055 3yr EFS 84.5% *vs.* 76.8% HR 0.63; *P* < 0.001
IMpassion-031 NCT03197935	III	Stage II/III TNBC	Chemotherapy +/- pembrolizumab	pCR 58% *vs.* 41%; *P* = 0.0044 (all-comers) pCR 69% *vs.* 49% *P* = 0.021 (significance boundary 0.0184) (PD-L1 + ve)
NeoTRIP NCT002620280	III	Early high-risk and locally advanced TNBC	Chemotherapy +/- atezolizumab followed by surgery, then adjuvant anthracyclines	pCR 48.6% *vs.* 44.4% OR 1.18; *P* = 0.48
GeparNuevo NCT02685059	II	Non-metastatic TNBC	Chemotherapy +/- durvalumab *window phase included 2 weeks of durvalumab/placebo	pCR 53.4% *vs.* 44.2% OR 1.45; *P* = 0.224 3 yr iDFS 84.9% *vs.* 76.9% HR 0.54; *P* = 0.0559 3 yr OS 95.1% *vs.* 83.1% HR 0.26; *P* = 0.0076

CPS: Combined positive score; EFS: event-free survival; ICI: immune checkpoint inhibitor; iDFS: invasive disease-free survival; mOS: median OS; ORR: objective response rate; OS: overall survival; pCR: pathologic complete response; PD-L1: programmed cell death ligand-1; PFS: progression-free survival; TNBC: triple-negative breast cancer.

In the adjuvant setting, there are ongoing trials such as the A-BRAVE trial^[46]^ investigating the use of avelumab in the treatment of high-risk TNBC, as well as the ALEXANDRA/IMpassion030 trial^[47]^ evaluating standard chemotherapy with or without atezolizumab in patients with early-stage TNBC. Additionally, the use of ICIs in early relapsing TNBC is also being investigated in the IMpassion 132 trial, a phase III randomised trial evaluating the role of combining atezolizumab with chemotherapy in patients with locally recurrent inoperable or metastatic TNBC within 12 months from receiving curative-intent treatment^[48]^.

### ICIs in other subtypes of breast cancer

While there have also been efforts to evaluate the use of ICIs in HER2-positive and hormone-positive/HER2-negative breast cancers, none of the studies have led to conclusive evidence for its use in these settings at present. In particular, HER2-positive breast cancer is thought to share certain similarities with TNBC that might suggest a benefit from ICI therapy. This includes the presence of higher tumour infiltrating lymphocytes (TILs) and PD-L1 expression. The presence of TILs in the tumour and its surrounding microenvironment is thought to be a reflection of pre-existing antitumour immunity^[49,50]^, and its presence is thought to be predictive of response to systemic anti-cancer treatment^[50]^, as well as a prognostic biomarker^[24]^. TNBC and HER2-positive breast cancers have been observed to have a higher number of TILs compared to hormone-positive breast cancers^[51,52]^. PD-L1 expression has also been observed to be upregulated in HER2-positive breast cancer^[53]^, and be predictive of response to ICIs in the PANACEA and KATE2 studies^[54,55]^. Further in-depth discussion of ICIs in HER2-positive and hormone-positive/HER2-negative breast cancers is beyond the scope of our current article, but has been extensively reviewed^[56-58]^.

## UNDERSTANDING AND OVERCOMING RESISTANCE MECHANISMS TO ICIS

Given that the earliest approval for ICI use in breast cancer came on 8 March 2019 for atezolizumab in combination with nab-paclitaxel in metastatic TNBC based on the IMpassion 130 trial^[33]^, the experience and evidence available on resistance mechanisms specific to immunotherapy in breast cancer is scarce. In addition, discounting tumour agnostic approvals, which form a very small proportion of breast cancer patients as discussed above^[36,37]^, the approval for ICIs in breast cancer is now only limited to the TNBC subtype, which constitutes only 15%-20% of all patients with breast cancer^[59]^, and even so, only a subset of them with high risk early-stage and metastatic disease. Hence, much of our understanding of resistance to ICIs comes from the available data and research on ICI treatment as a whole from various other tumour types.

**Figure 1:**
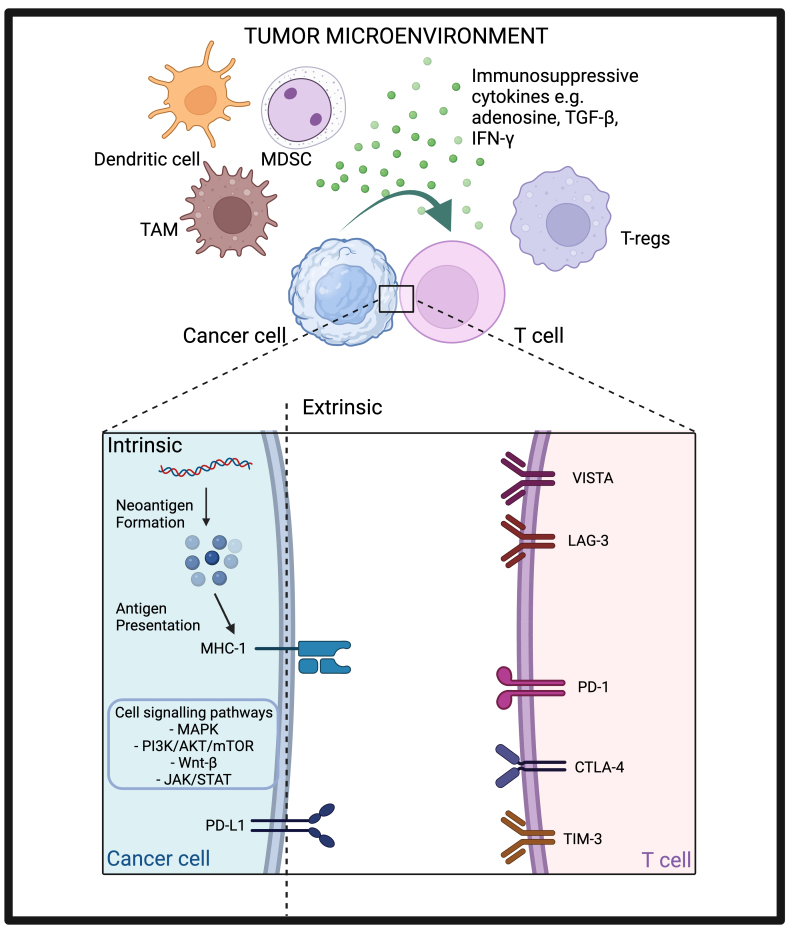
Tumour intrinsic and extrinsic resistance pathways to ICIs. Created with BioRender.com. AKT: Protein kinase B; CTLA-4: cytotoxic T-lymphocyte-associated antigen 4; IFN-γ: interferon-γ; JAK: janus kinase; LAG-3: lymphocyte-activation gene 3; MAPK: mitogen-activated protein kinase; MDSC: myeloid-derived suppressor cell; MHC-I: major histocompatibility complex-I; mTOR: mammalian target of rapamycin; PD-1: programmed cell death protein-1; PD-L1: programmed cell death ligand-1; PI3K: phosphatidylinositol 3-kinase; STAT: signal transducers and activators of transcription; TAM: tumour-associated macrophage; TGF-β: transforming growth factor-β; TIM-3: T-cell immunoglobulin, mucin domain-3 protein; T-reg: regulatory T cell; VISTA: V-domain immunoglobulin suppressor of T-cell activation.

Resistance pathways to ICIs can be tumour-intrinsic, e.g., alteration of certain genes or signalling pathways within the tumour, or tumour-extrinsic, e.g., changes in components within the tumour microenvironment (TME) other than the tumour cell itself^[60]^. This can happen either from the outset, conferring primary resistance whereby no response to treatment is noted, or after a period of observed response, highlighting the concept of acquired resistance. As previously mentioned, breast cancers are known to be immunogenically cold tumours, which contributes to their primary resistance to ICI. We will discuss the various mechanisms of resistance by looking at both tumour-intrinsic and tumour-extrinsic pathways, and how each of them might potentially be harnessed to overcome drug resistance [Figure 1].

## TUMOUR INTRINSIC RESISTANCE MECHANISMS TO ICIS

### Alteration of signalling pathways

There are several critical signalling pathways that control cell-cycle progression, apoptosis, and cell growth. Alterations in any of these pathways can sometimes be exploited by cancer cells to escape immune surveillance, leading to resistance to ICIs. Some of these pathways are known to be more commonly mutated in breast cancer, for example, the mitogen-activated protein kinase (MAPK) pathway, phosphatidylinositol 3-kinase (PI3K)/protein kinase B (AKT)/mammalian target of rapamycin (mTOR) pathway, Wnt/β-catenin pathway and Janus kinase (JAK) and signal transducers and activators of transcription (STAT) pathway^[61]^. Hence, various combination therapies of ICIs with other therapeutic agents to target each of these specific pathways are gaining traction and have shown promising preliminary activity.

#### MAPK pathway

Signalling via the MAPK pathway induces the expression of various proteins such as vascular endothelial growth factor (VEGF) as well as interleukin (IL)-8 that inhibit T cell recruitment and function^[62]^. Inhibiting the MAPK pathway can also upregulate major histocompatibility complex (MHC)-I, MHC-II, and PD-L1 expression, and enhance infiltration of TILs^[63]^. Loi *et al.* had confirmed this observation in an analysis of 111 patients with TNBC who had been treated with neoadjuvant chemotherapy, and demonstrated that alterations in the MAPK signalling pathway can suppress the expression of both MHC-I and MHC-II^[64]^.

Hence, trials evaluating the combination of mitogen-activated protein kinase kinase (MEK or MAP2K) inhibitors with ICIs are ongoing. The COLET trial^[65]^ was a phase II trial that investigated cobimetinib, a MEK inhibitor, in combination with atezolizumab and taxane chemotherapy in untreated metastatic TNBC. It demonstrated a numerical but nonsignificant increase in ORR of 34.4% and 29% in patients treated with paclitaxel *vs.* nab-paclitaxel, respectively. Exploratory biomarker analysis suggested that patients with PD-L1-positive disease (defined as IC ≥ 1% by the SP142 IHC assay) had numerically higher ORR compared to those with PD-L1 negative disease (39% *vs.* 19%), as well as median PFS (7.0 *vs.* 3.7 months).

#### PI3K/AKT/mTOR pathway

Abnormalities in the PI3K/AKT/mTOR pathway are also another well-known mechanism of resistance in breast cancer^[66]^. The protein phosphatase and tensin homolog (PTEN) tumour suppressor is a negative regulator of PI3K signalling and deletions in PTEN result in the enhancement of PI3K signalling^[66,67]^. PTEN loss has also been associated with resistance to T cell-mediated immunotherapy by increasing the expression of immunosuppressive cytokines, particularly VEGF^[68]^. VEGF can contribute further to immunosuppressive TME by recruiting suppressive immune cells such as myeloid-derived suppressor cells (MDSC) and regulatory T cells (Tregs)^[69]^.

Based on these preclinical findings, AKT inhibitors have been combined with ICIs to overcome this resistance pathway. The phase Ib study evaluating the triplet combination of ipatasertib, atezolizumab, and a taxane as first-line treatment for locally advanced/metastatic TNBC reported a promising ORR of 73% irrespective of their PD-L1 status or PIK3CA/AKT1/PTEN alteration status^[70]^. The BEGONIA study (NCT03742102) is a phase Ib/II trial evaluating the combination of durvalumab with different novel oncologic therapies designed for immune modulation, with or without paclitaxel as first-line treatment in patients with metastatic TNBC. In arm 2^[71]^, the addition of capivasertib was studied, yielding an ORR of 53.3%. Importantly, there was a relatively high rate of G3/4 treatment-related adverse events of 73%, although only 6.7% discontinued treatment due to adverse events.

#### Wnt/*β*-catenin pathway

The Wnt/β-catenin pathway is an important oncogenic signalling pathway involved in many essential cellular processes^[72]^. The activation of Wnt results in the accumulation of the transcriptional co-activator β-catenin to initiate the transcription of several cell cycle genes and oncogenes such as Myc^[73]^. The high levels of β-catenin via the canonical pathway have also been shown in a murine study by Spranger *et al.* to decrease the presence of CD103+ dendritic cell (DC) by reducing the expression of chemokine that attracts CD103+ DC (CCL4), preventing the migration of DC into the TME^[74]^. Consequently, this results in the blocking of adaptive antitumour immunity^[75]^. A study of TNBC by Castagnoli *et al.* showed that TNBC stem cells are able to upregulate PD-L1 expression via the Wnt pathway^[75]^.

#### JAK/STAT pathway

Interferon γ (IFN-γ) is a cytokine produced by activated T cells and antigen-presenting cells (APCs) that is critical in immune cell activation via the Janus kinase 1 and 2 (JAK1/2) as well as signal transducers and activators of transcription-1 (STAT1) pathway^[76]^. Any mutation or epigenetic silencing of molecules in this pathway allows tumours to escape its apoptotic or cytostatic effect^[77]^. A study analysing melanoma patients who were treated with ICI therapy and subsequently developed resistance noted that resistance was associated with defects such as loss-of-function mutations in the JAK1/2 pathway^[78]^. Another study of 16 melanoma patients observed that those who were non-responders to CTLA-4 inhibition harbor a much higher rate of genomic changes in the IFN-γ pathway genes compared to those who responded^[79]^.

### Antigen presentation

A crucial feature of adaptive immunity is its ability to recognise antigens that are foreign or not “self”. Cancer cells generally harbour accumulated somatic mutations and genomic instability within DNA coding regions. Antigen peptide sequences that distinguish tumour cells are classified based on their unique cell expression patterns^[73]^. Tumour-specific antigens (TSA) refer to novel peptide sequences, i.e., neo-antigens, that develop via mutations and are not present in normal healthy cells. Examples of mutations that result in TSAs usually involve oncogenic driver mutations, such as mutations in the *BRCA1/2* gene^[80]^. The presentation of these neoantigens by APCs via MHC-I molecules is critical in priming specific cytotoxic CD8+ T cells, thereby triggering an immune response towards the tumour. Indeed, studies have shown that increasing neoantigen formation helps to improve response to ICIs^[81-83]^.

#### Antibody-drug conjugates

Similar to the rationale for combining ICIs with chemotherapy which was discussed earlier, antibody-drug conjugates (ADCs) also increase tumour neoantigen formation via immunogenic cell death^[84]^. An ADC consists of an antigen-specific monoclonal antibody bound to a cytotoxic payload via a molecular linker. The binding of an ADC via its antigen-binding portion induces its internalisation via endocytosis. Once inside the tumour cell, cleavage of its linker through proteolysis results in the release of the cytotoxic payload. This allows for target-dependent activation and selective cytotoxicity^[85]^. Of note, two ADCs, namely trastuzumab deruxtecan (T-DXd) and sacituzumab govitecan, have received FDA approvals for the treatment of specific breast cancer subtypes. T-DXd is approved for unresectable or metastatic HER2-positive breast cancer based on the results of DESTINY-Breast 03, confirming significant PFS benefit (HR 0.28; *P* < 0.0001)^[86]^, as well as for unresectable or metastatic HER2-low breast cancer based on DESTINY-Breast 04 showing both promising PFS (HR 0.50; *P* < 0.001) and OS (HR 0.64; *P* = 0.001) benefit^[87]^. Sacituzumab govitecan, on the other hand, has been approved both for unresectable or metastatic TNBC as well as hormone-positive, HER2-negative breast cancer based on the ASCENT and TROPiCS-02 trials, respectively, both confirming PFS and OS benefit^[88,89]^.

Preclinical data have suggested that the combination of ADCs with ICIs may improve the efficacy of ICIs via increasing neoantigen formation and presentation, as well as by activating DCs and increasing the expression of PD-L1^[85]^. There are currently several ongoing trials evaluating the combination of different ADCs with ICIs. In the earlier described BEGONIA study, two ADCs, T-DXd and datopotamab deruxtecan (Dato-DXd), are being studied in arms 6 and 7 of the trial, respectively. Preliminary data for both arms were promising; ORR with the addition of T-DXd was 66.7%^[90]^ and 74% with the addition of Dato-DXd^[91]^. Other ongoing trials in this space are summarised in Table 2.

**Table 2:** Summary of ongoing trials evaluating the addition of ADC to ICI therapy

**Trial name/ID**	**Phase**	**Patients enrolled**	**ICI**	**ADC**	**Primary endpoint(s)**
ASCENT-04 NCT05382286	III	Treatment naïve advanced/metastatic TNBC	Pembrolizumab	Sacituzumab Govitecan	PFS
NCT04448886	II	Metastatic HR+/HER2- breast cancer who have progressed on or within 12 months of adjuvant endocrine or ≥ 1 endocrine therapy in the metastatic setting	Pembrolizumab	Sacituzumab Govitecan	PFS
NCT03310957	I/II	Advanced/Metastatic TNBC	Pembrolizumab	SGN-LIV1A	ORR, DLT, adverse events
Morpheus-TNBC NCT03424005	Ib/II	Metastatic TNBC	Atezolizumab	Sacituzumab Govitecan or SGN-LIV1A	ORR, adverse events
InCITe NCT03971409	II	Metastatic TNBC	Avelumab	Sacituzumab Govitecan	ORR
Astefania NCT04873362	III	Patients with residual invasive disease in breast/axillary lymph nodes following neoadjuvant chemotherapy	Atezolizumab	Trastuzumab emtansine	Invasive disease-free survival
KATE3 NCT04740918	III	Metastatic PD-L1-positive cancer after progression on H +/- P and taxane	Atezolizumab	Trastuzumab emtansine	PFS, OS
NCT03032107	I	Metastatic breast cancer on progression on prior H and a taxane	Pembrolizumab	Trastuzumab emtansine	Safety and tolerability
NCT04042701	Ib	Metastatic HER2 positive or HER2 low breast cancer	Pembrolizumab	Trastuzumab deruxtecan	DLT and ORR
NCT03523572	I	Metastatic breast cancer progressed on ≥ 2 anti-HER2-based regimens	Nivolumab	Trastuzumab deruxtecan	DLT, ORR
DESTINY-Breast07 NCT04538742	Ib/II	Metastatic 2nd line and beyond (Part 1) and 1st line (Part 2)	Durvalumab	Trastuzumab deruxtecan	Safety and toxicity
DESTINY-Breast08 NCT04556773	I	Advanced or metastatic HER2-low breast cancer	Durvalumab	Trastuzumab deruxtecan	Safety and toxicity

ADC: Antibody-drug conjugate; DLT: dose-limiting toxicities; ICI: immune checkpoint inhibitor; ORR: objective response rate; OS: overall survival; PD-L1: programmed cell death ligand-1; PFS: progression-free survival; TNBC: triple-negative breast cancer.

#### Poly(ADP-ribose) polymerase inhibitors

Poly(ADP-ribose) polymerase (PARP) inhibitors increase DNA damage, leading to more TSAs and also increased MHC-I expression, thereby causing increased antigen presentation^[92]^. The increase in DNA damage associated with breast cancer patients who harbour the *BRCA1/2* mutation occurs via a process known as synthetic lethality^[93]^. The use of PARP inhibitors blocks the repair of single-stranded DNA breaks via base excision repair. This allows single-stranded breaks to accumulate, leading to the generation of double-stranded breaks (DSBs). These DSBs can usually be restored by either the high-fidelity homologous repair pathway or the error-prone non-homologous end-joining method. As *BRCA1/2* mutant breast cancer patients already have existing defects in homologous repair, they are unable to effectively repair DNA damage, resulting in the generation of TSAs. In addition to increasing antigen presentation, PARP inhibitors have also been shown in preclinical studies to alter the TME by activating intra-tumoural dendritic cells and increasing CD8+ T cell infiltration via the STING (stimulator of interferon genes) pathway^[94]^. It also enhances the upregulation of PD-L1 expression by reducing the PARylation of STAT3^[95]^. The latter two mechanisms help to overcome tumour extrinsic mechanisms of resistance to immunotherapy that will be expounded upon later.

Consequently, there have been several studies evaluating the combination of PARPi together with ICIs. The TOPACIO/KEYNOTE-162 trial^[96]^ studied the efficacy of niraparib together with pembrolizumab in 55 patients with metastatic TNBC. In the subgroup of patients with *BRCA1/2* mutations, the ORR was 47% and mPFS 8.3 months. In contrast, patients who were non-*BRCA1/2* mutants had an ORR of 11% and mPFS of 2.1 months. The MEDIOLA trial^[97]^ studied the combination of olaparib and durvalumab as first or second-line therapy in germline *BRCA1/2* mutant metastatic TNBC, noting an ORR of 63%, mPFS of 8.2 months and mOS 21.5 months. Table 3^[96-101]^ summarises some of the available trials evaluating this combination.

**Table 3:** Summary of ongoing trials evaluating the addition of PARPi to ICI therapy

**Trial name/ID**	**Phase**	**Patients enrolled**	**ICI**	**PARPi**	**Primary endpoint(s)**	**Results (if any)**
TOPACIO/ KEYNOTE-162 NCT02657889	I/II	Advanced or metastatic TNBC	Pembrolizumab	Niraparib	DLT and ORR	ORR 21%, 47%, 11% (overall, BRCA mutant, BRCA wild-type)^[96]^
NCT04683679	II	Metastatic TNBC or HR+/HER2- breast cancer	Pembrolizumab	Olaparib	ORR	
NCT03101280	Ib	Previously treated metastatic TNBC with BRCA mutation or BRCA-like molecular signature	Atezolizumab	Rucaparib	Number of dose modifications due to adverse events	
NCT02849496	II	Advanced or metastatic non-HER2-positive breast cancer with homologous DNA repair deficiency	Atezolizumab	Olaparib	PFS	
NCT04690855	II	Germline BRCA1/2 negative, PD-L1 positive metastatic TNBC	Atezolizumab	Talazoparib	ORR	
MEDIOLA NCT02734004	I/II	Germline BRCA mutated metastatic HER2-negative breast cancer	Durvalumab	Olaparib	DCR, safety, and tolerability	DCR at 12 weeks 80%, 28 weeks 50% ORR 63.3%^[97]^
DORA NCT03167619	II	Platinum-treated metastatic TNBC	Durvalumab	Olaparib	PFS	Combination arm: mPFS 6.1 mo, DCR 68.2%^[98]^
DOLAF NCT04053322	II	Advanced ER+, HER2- breast cancer with BRCA mutation, alteration in homologous recombination repair or MSI	Durvalumab	Olaparib	PFS	
PHOENIX NCT03740893	II	Post-neoadjuvant chemotherapy with residual TNBC	Durvalumab	Olaparib	Biomarker study pre-surgery and post-surgery	
NCT03801369	II	Metastatic TNBC	Durvalumab	Olaparib	ORR	
NCT03544125	I	Metastatic TNBC	Durvalumab	Olaparib	Safety and efficacy	
NCT02484404	I/II	Advanced TNBC	Durvalumab	Olaparib	Dose finding and toxicities	
JAVELIN PARP Medley NCT03330405	Ib/II	Advanced/ metastatic TNBC or HR+/HER2- breast cancer	Avelumab	Talazoparib	DLT and ORR	ORR 18.2% and 34.8% (TNBC, HR+/HER2-)^[99]^
JAVELIN BRCA/ATM NCT03565991	II	BRCA or ATM mutant advanced or metastatic solid tumour	Avelumab	Talazoparib	ORR	ORR 26.4% (BRCA) 4.9% (ATM)^[100]^
TALAVE NCT03964532	I/II	Advanced breast cancer	Avelumab	Talazoparib	Safety and toxicities	
NCT03945604	Ib	Recurrent, metastatic TNBC	Camrelizumab (anti-PD-1)	Fluzoparib	DLT	mPFS 5.2 mo, 12 mo OS 64.2%^[101]^

DCR: Disease control rate; DLT: dose-limiting toxicities; ICI: immune checkpoint inhibitor; MSI: microsatellite instability; ORR: objective response rate; OS: overall survival; PARPi: poly(ADP-ribose) polymerase inhibitors; PD-L1: programmed cell death ligand-1; PD-1: programmed cell death protein-1; PFS: progression-free survival; TNBC: triple-negative breast cancer.

Tumour cells can also evade immune surveillance by altering any step in the antigen presentation pathway, thereby conferring resistance to treatment with ICIs. Several studies involving patients with breast cancer have reported the downregulation of expression of the transporters TAP1, TAP2, and TAPBP, which are necessary for transporting antigens to be loaded onto MHC molecules^[102-104]^. Other mechanisms that have been observed include loss of heterozygosity and epigenetic suppression of certain MHC-I molecules^[105]^ or alterations in the expression of beta-2-microglobulin (B2M) which is essential for the transport and subsequent expression of MHC-I on the cell surface^[105,106]^. Luo *et al.* reported the potential use of DNA methyltransferase inhibitors to overcome resistance to immunotherapy in breast cancer patients^[107]^.

## TUMOUR EXTRINSIC MECHANISMS OF RESISTANCE TO IMMUNOTHERAPY

### Alteration of the tumour microenvironment

The TME comprises various components that are constantly evolving, with ongoing crosstalk between tumour and stromal cells, all of which can influence the immune response and drive resistance to ICIs^[73]^. The presence of TILs in the tumour and its surrounding microenvironment is thought to be a reflection of pre-existing antitumour immunity^[49,50]^, and its presence is thought to be predictive of response to systemic anti-cancer treatment^[50]^, and a prognostic biomarker^[24]^. TNBC and HER2-positive breast cancers have a higher number of TILs^[51,52]^. Other components of the TME include Tregs, MDSCs, tumour-associated macrophages (TAMs), and cytokines.

Tregs suppress effector T cells and APC via secretion of inhibitory cytokines, direct contact, and limiting inflammation^[108]^. The increased infiltration of Tregs into tumour cells has been observed in several other tumour types^[109,110]^, and murine studies have demonstrated that depleting Tregs from the TME can help to restore antitumour immunity^[109]^.

The presence of MDSCs in the TME has also been shown to promote angiogenesis, immune evasion, tumour growth and metastasis^[108]^. A study of patients with melanoma treated with CTLA-4 inhibitors suggested that the increase in MDSCs was associated more often with non-responders^[111]^. Interestingly, the γ isoform of PI3K has been noted to be highly expressed in MDSC cells in a study of several cancer types, including breast cancer^[112]^, and selectively inhibiting it can help to re-establish sensitivity to ICIs^[113]^.

Another important group of cells present in the TME that promote immunosuppression and play a role in resistance to immunotherapy are TAMs, which consist of M1 and M2 macrophages^[114]^. M1 macrophages are mainly involved in antitumour immunity, while M2 macrophages are pro-tumourigenic. The accumulation of TAMs is regulated by cytokines, such as chemokine ligand 2 (CCL2), which was demonstrated by Qian *et al.* in their study using breast cancer- bearing murine model^[115]^, as well as colony-stimulating factor-1 (CSF-1). It was observed to be correlated with increased macrophage infiltration and more frequent metastases in breast cancer patients^[116]^. Indeed, studies that evaluated CSF-1 receptor inhibition in combination with ICI treatment showed synergy of both agents and promising tumour regression, suggesting that CSF-1 receptor inhibitors can help to overcome tumour resistance to immunotherapy^[117,118]^.

Besides individual populations of cells, the make-up of various cytokines present in the TME is also important in immune cell recruitment, activation, and proliferation by its balance of both stimulatory and suppressive effects^[119]^. For example, cytokines such as transforming growth factor β (TGF-β) induce immunosuppression by upregulating Tregs and inhibiting cytotoxic T lymphocytes^[120]^. Tumour cells also express ecto-5’-nucleotidase (CD73), which is an enzyme that dephosphorylates adenosine monophosphate (AMP), forming adenosine^[121]^. Adenosine is a potent immunosuppressor that binds to A2A receptors found on lymphocytes and suppresses its function^[122]^. Breast cancer cells have been shown to express CD73^[123]^, and its expression appears to be regulated by the estrogen receptor (ER), whereby the loss of ER enhances the expression of CD73^[124]^. A proof of concept study confirmed that anti-CD73 antibody therapy can trigger adaptive antitumour immunity and inhibit metastasis in breast cancer^[125]^.

### Upregulation of other immune checkpoints

Resistance to ICIs can also be achieved via upregulation of other immune checkpoints such as T-cell immunoglobulin, mucin domain-3 protein (TIM-3), LAG-3, V-domain immunoglobulin suppressor of T-cell activation (VISTA), B and T lymphocyte attenuator (BTLA), and T-cell immunoreceptor tyrosine-based inhibition motif domain (TIGIT)^[108,126-128]^. The co-expression of multiple immune checkpoints has been demonstrated to be associated with T cell exhaustion, and subsequently resistance to ICIs^[129]^. Targeting these alternative pathways represents potential therapeutic options for overcoming drug resistance to ICIs. Although most studies evaluating such combination strategies have been in other tumour types such as melanoma and NSCLC^[130-132]^, these are still relevant in breast cancers as epigenetic modifications resulting in upregulation of multiple immune checkpoints such as PD-L1, CTLA-4, TIM-3, and LAG-3 have been observed, and correlated with poorer patient prognosis in a study of breast cancer patients^[133]^. A study that specifically included breast cancer patients was a phase I study of LAG525, a monoclonal antibody blocking the binding of LAG-3 to MHC-II in combination with spartalizumab (an anti-PD-1 antibody) in patients with advanced malignancies, which showed durable responses^[134]^. In particular, 2 out of 5 patients with advanced TNBC showed a response, and in TNBC tumour biopsies, a trend in the conversion of immune-cold to immune-activated biomarker profiles was reported^[134]^.

## CONCLUSION: CHALLENGES AND FUTURE DIRECTIONS

Aside from resistance mechanisms to ICIs, there are also many unresolved and unanswered questions that have limited the use of ICIs in breast cancer. These include identifying the best predictive and prognostic biomarkers to guide treatment, evaluating the optimal duration of ICIs in the neoadjuvant/adjuvant setting, and chemotherapy backbone in the metastatic setting, just to name a few. Recent review articles have discussed some of these topics^[135,136]^.

Further advancement in this field needs to be led by sound science with good preclinical evidence from appropriate murine tumour models that can reflect the human immune environment. While this has conventionally largely been restricted due to a limited selection of murine tumour models, novel syngeneic tumour murine models have been better able to mirror the genomic heterogeneity of human cancer, and recapitulate the TME so as to provide accurate results. It is hoped that the use of appropriate novel syngeneic tumour murine models will allow us to further study ICI combinations effectively and accurately^[137]^.

Lastly, studies looking beyond immunotherapy-based treatments are also being investigated. One such area is the study of the human gut microbiome, a host factor that influences not only the biology of tumour development but also the modulation of its response and resistance to immunotherapy^[138-140]^. Consequently, there are ongoing studies looking at modifying the gut microbiota in order to increase the efficacy of immunotherapy treatment. These include interventions such as the use of antibiotics, probiotics, faecal microbiota transplantation, and diet and prebiotics^[141]^.

There is much to be anticipated in this evolving field of immunotherapy in breast cancer. While previously thought to be an immunologically “cold” cancer with limited responses to ICI, this is certainly set to change. The numerous ongoing trials evaluating ICIs in combination with novel therapies to overcome resistance and exploit the immune system, as well as the development of innovative immunomodulatory strategies, will allow us to further harness and expand the role of immunotherapy in breast cancer.

## DECLARATIONS

### Copyright

© The Author(s) 2023.
